# Deng Entropy Weighted Risk Priority Number Model for Failure Mode and Effects Analysis

**DOI:** 10.3390/e22030280

**Published:** 2020-02-28

**Authors:** Haixia Zheng, Yongchuan Tang

**Affiliations:** School of Big Data and Software Engineering, Chongqing University, Chongqing 401331, China; 20171766@cqu.edu.cn

**Keywords:** failure mode and effects analysis (FMEA), risk priority number (RPN), Dempster–Shafer evidence theory (DST), Deng entropy, uncertainty management

## Abstract

Failure mode and effects analysis (FMEA), as a commonly used risk management method, has been extensively applied to the engineering domain. A vital parameter in FMEA is the risk priority number (RPN), which is the product of occurrence (O), severity (S), and detection (D) of a failure mode. To deal with the uncertainty in the assessments given by domain experts, a novel Deng entropy weighted risk priority number (DEWRPN) for FMEA is proposed in the framework of Dempster–Shafer evidence theory (DST). DEWRPN takes into consideration the relative importance in both risk factors and FMEA experts. The uncertain degree of objective assessments coming from experts are measured by the Deng entropy. An expert’s weight is comprised of the three risk factors’ weights obtained independently from expert’s assessments. In DEWRPN, the strategy of assigning weight for each expert is flexible and compatible to the real decision-making situation. The entropy-based relative weight symbolizes the relative importance. In detail, the higher the uncertain degree of a risk factor from an expert is, the lower the weight of the corresponding risk factor will be and vice versa. We utilize Deng entropy to construct the exponential weight of each risk factor as well as an expert’s relative importance on an FMEA item in a state-of-the-art way. A case study is adopted to verify the practicability and effectiveness of the proposed model.

## 1. Introduction

Risk represents the possibility of unintended faults occurring in a system. Uncertainty is a key issue in risk analysis and management [1,2]. Since it was introduced by NASA in 1960s [3], failure mode and effects analysis (FMEA), as a typical bottom-up technique to model and manage potential risks [4], has been extensively applied to various industries, such as medical domain [5,6,7], aircraft landing system [8], assisted reproduction technology [9], automotive industry [10] and so on [11]. In these piratical applications, FMEA is mainly used to ensure that potential risks have been taken into account and dealt with properly during the assessment process. Its most visible result is the documentation of the collective knowledge of cross-functional teams [12]. Traditionally, in this method, risk priority number (RPN) is generally adapted to evaluate and rank the potential failure modes. RPN is a product of three risk factors in FMEA: the probability of occurrence of a failure mode (*O*), the severity of a failure effect (*S*), and the probability of a failure being detected (*D*), which already have been involved in risk assessments. The various components of the target system are prioritized based on RPN scores [13].

Traditional FMEA processes can be summarized as follows, (1) determine the scope of FMEA and assemble a team, (2) identify potential failure failure modes and effects, (4) calculate the RPN of each failure mode and (5) prioritize the failure modes and report the analysis results [3]. Among the processes of the FMEA approach, due to the increasing complexity of system or process, there exists uncertainty when FMEA team members provide their judgments on the identified failure modes. Furthermore, the conventional RPN model is sometimes not that efficient in practical applications [14,15,16]. Flaws of classical RPN can be concluded from three aspects [17,18]. First and foremost, different values of *O*, *S*, and *D* multiplied may be the same RPN value, although they have completely different meanings of risk. For instance, an expert evaluates two different failure modes as (8, 1, 3) and (1, 8, 3) for (*O*, *S*, and *D*), respectively. Though their RPN values are exactly the same, the severity and occurrence of failure 1 is obviously different from that of failure 2. Second, the conventional RPN model ignores the relative importance among *O*, *S*, and *D*. Nevertheless, when applying FMEA this may not be the case with the same weight for the three risk factors. Last but not least, according to the suggested ratings in [19], the RPN score is between 1 and 1000 because each risk factor is on a scale of 1 to 10. However, only 120 of the 1000 numbers can be generated for the product of three risk factors.

Some improved methods have been presented to resolve the aforementioned problems. The uncertainty in complex systems, part of which comes from domain experts’ subjective opinions, needs to be modeled by a reasonable method. In addition to FMEA processes, uncertain information can be processed in practical applications such as distribution networks [20], Mechatronics Engineering [21], nuclear industry [22], infomation fusion and optimization [23], and so on [24]. To deal with the fuzziness in information processing, many theories and methods have been presented, such as Dempster–Shafer evidence theory (DST) [25,26], probability theory [27,28], information entropy [29], fuzzy sets theory [30], and so on [31,32]. For example, in [33], a new risk measure combined with information entropy to measure risk in clustering is proposed. In [34], a measure for modelability, the degree to which an uncertain or fuzzy parameter can be modeled, is presented. Similarly, gray relational projection [35], TOPSIS (technique for order preference by similarity to an ideal solution) [36] and TODIM (an acronym in Portuguese of interactive and multicriteria decision making) [15,37] are utilized to deal with the uncertainty and fuzziness. Because of its capability in uncertain information fusion [38,39,40], DST is used to manage uncertain information in this paper.

Deng entropy, first proposed in [41], aims to measure the uncertain degree of basic probability assignment (BPA) in the framework of DST. Compared with the measure of aggregate uncertainty (AU) [42,43], the ambiguity (AM) [44], and other existing methods for modeling uncertainty, Deng entropy has some advantages [41]. First, Deng entropy satisfies some of the axiomatic requirements that have been further built in order to develop a justifiable measure. The five requirements include range, probabilistic consistency, set consistency, additivity, and subadditivity [45]. Second, Deng entropy is the generalization of Shannon entropy in DST; it provides a promising way to measure uncertain degree and to handle more uncertain information. Third, it is proved that Deng entropy increases monotonously with the increase of the size in a proposition, which also shows it can change more sensitively and rationally than other uncertainty measures. In addition, Deng entropy handles some problems in some other measures in the DST framework [41]. Thus, in this paper, Deng entropy is adopted to measure the uncertainty of experts’ judgments in the framework of DST.

From the perspective of overcoming the shortcomings in traditional RPN, the mean value of RPN (MVRPN) [46], the generalized evidential RPN (GERPN) [47], ambiguity measure weighted risk priority number (AMWRPN) [48], and some other methods [49,50,51] are proposed. For example, in [48], AMWRPN model for FMEA is presented. It processes the weights of risk factors utilizing the fuzziness of experts’ evaluations. Furthermore, fuzzy set theory is an efficient approach to model uncertainty assessments [52,53,54,55]. In [56], the defuzzification of triangular fuzzy numbers is used to determine RPN. In [54], a novel FMEA model utilizing linguistic terms and components’ weights is proposed. In [57], a 2-tuple linguistic variables structure and gray relational analysis are combined to process various assessments of FMEA team members. In addition, in [58], cloud model theory and TOPSIS method are utilized to deal with the risk factor assessments given as linguistic values. However, the previous methods do not take the relative importance of both risk factors and different experts into account. Hence, to resolve this problem, a novel Deng entropy weighted RPN (DEWRPN) model for FMEA is proposed.

In this method, Deng entropy is firstly used to calculate the weights among three risk factors according to the assessments for every failure mode. Then, calculate the weight of each expert by using the corresponding three risk factors’ uncertainty measure-based weights. Finally, calculate the novel RPN based on a novel formula integrating two aforementioned weights. It should be noted that, in comparison with other improved FMEA approaches, there are three desirable properties of the proposed method. First, Deng entropy, as a generalization of Shannon entropy, is an efficient method to obtain the uncertain degree of BPA. Second, the proposed DEWRPN model considers the relative importance of both risk factors and experts, which is more compatible to the real decision-making situation. Last but not least, the value difference of the decision value obtained in this paper is larger than that in [54,59], making the failure mode easier to identify, and the method in this paper is more applicable in complex systems with high requirements for accuracy, which also contributes to decreasing the duplicate value of RPN.

The rest of this paper is organized as follows. The preliminaries are introduced in Section 2. In Section 3, a new Deng entropy weighted risk priority number approach for FMEA model, named DEWRPN, is proposed. Then, the DEWRPN-based FMEA approach is used to analyze a case study of steel production process in a steel factory in Section 4. The conclusion of this paper is shown in Section 5.

## 2. Preliminaries

In this section, some basic concepts in the DST [25,26], FMEA [3], and Deng entropy [41] are introduced.

### 2.1. Dempster–Shafer Evidence Theory

DST originated in the work of Dempster using probabilities with upper and lower bounds [25] and Shafer established the basic probability assignment function (BPA) on the framework of discernment [26]. DST develops on the foundation of the Bayesian theory of probabilities. It can represent and process uncertain information effectively. Reasoning and decision-making can be carried out with incomplete or conflicting pieces of evidence even if there is a lack of prior information. Formally, the definitions in DST are provided as follows.

**Definition** **1.** *Let* Ω *be a set of mutually exclusive and collectively exhaustive elements $H_{i}$, indicated by*

(1) $$\Omega =\left\{H_{1},H_{2},\ldots ,H_{i},\ldots ,H_{N}\right\}.$$

*The power set of* Ω *composed with $2^{N}$ propositions is called the Frame of Discernment (FOD), denoted as $2^{\Omega }$:*

(2) $$2^{\Omega }=\left\{\begin{array}{c} \emptyset ,\left\{H_{1}\right\},\left\{H_{2}\right\},\ldots ,\left\{H_{N}\right\},\left\{H_{1},H_{2}\right\},\\ \ldots ,\left\{H_{1},H_{2},\ldots ,H_{i}\right\},\ldots ,\Omega \end{array}\right\},$$

*where each element is a proposition and* ∅ *is an empty set. In addition, each single set that contains only one element in FOD is called singleton.*

**Definition** **2.** *A basic probability assignment (BPA) (also called mass function) is a mapping for elements in $2^{\Omega }$ to the interval [0,1], formally defined by:*

(3) $$m:2^{\Omega }\to \left[0,1\right],$$

*which satisfies the following conditions:*

(4) $$m\left(\emptyset \right)=0,\sum_{A\in \Omega }m\left(A\right)=1,$$

*where “A” symbolizes any subset of* Ω*, which is A⊆Ω. If A≠∅, the BPA function m(A) represents how strongly the evidence supports the hypothesis A. If m(A)>0, the A in the frame of discernment is called a focal element and the set of all the focal elements is named a body of evidence (BOE).*

**Definition** **3.** *A BPA m can also be represented by the belief function Bel or the plausibility function Pl, defined as follows:*

(5) $$Bel\left(A\right)=\sum_{\emptyset \neq B\subseteq A}m\left(B\right)\;,\;Pl\left(A\right)=\sum_{B\cap A\neq \emptyset }m\left(B\right).$$

**Definition** **4.** *Two pieces of evidence in the frame of discernment* Ω *indicated as $m_{1}$ and $m_{2}$. A focal elements of $m_{1}$ is described as B and that of $m_{2}$ is presented as C. The Dempster’s combination rule can be defined as follows:*

(6) $$\begin{array}{cc} m_{1,2}\left(A\right)= & m_{1}\left(B\right)⊕m_{2}\left(C\right)\\ & =\frac{\sum_{B,C\in \Omega ,B\cap C=A}m_{1}\left(B\right)\times m_{2}\left(C\right)}{1-\sum_{B\cap C=\emptyset ,B\cap C=A}m_{1}\left(B\right)\times m_{2}\left(C\right)}, \end{array}$$

*where a coefficient K is defined as follows:*

(7) $$K=\sum_{B\cap C=\emptyset }m_{1}\left(B\right)\times m_{2}\left(C\right).$$

Sometimes, the *K* is defined as a conflict coefficient between two BOEs.

**Definition** **5.** *Let m be a BPA defined on the frame of discernment X. The pignistic probability distribution $BetP_{m}$, called as such by Smets and Kennes [60], is defined for all the subsets of X by*

(8) $$BetP_{m}\left(A\right)=\sum_{B\subseteq X}m\left(B\right)\frac{\left|A\cap B\right|}{\left|B\right|},$$

*where |A| is the cardinality of set A.*

### 2.2. FMEA

**Figure 1:**
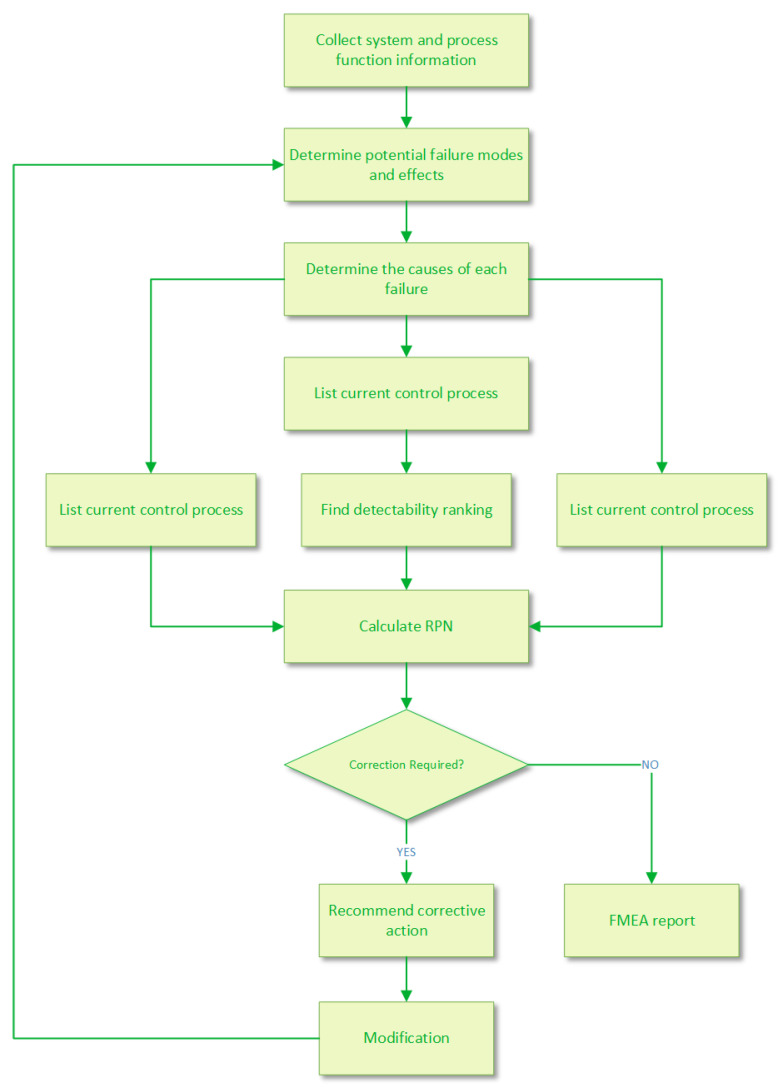
The flowchart of failure mode and effects analysis (FMEA).

FMEA is an analytical tool in reliability and risk management, which has been proved to be remarkably effective and applied extensively in many fields such as risk evaluation [58], decision making [61,62,63], and so on. A series of failure modes with high priorities are determined in conventional FMEA [64]. Facilitating the identification of potential failures in the design or process of products or systems, the process of applying an FMEA can be briefly concluded in Figure 1, where the FMEA process is summarized in detail as eleven steps [65].

- Step 1. Understand the operating properly process of a system.
- Step 2. Subdivide the system as subsystems and/or assemblies according to system’s characteristics.
- Step 3. Use various analysis tools to identify system’s components and how they relate to each other.
- Step 4. List all components for each subsystem and/or assembly.
- Step 5. Identify the key point that may have an impact on the performance of components and cause unexpected failures.
- Step 6. Determine the failure modes in detail for evaluation.
- Step 7. Domain experts evaluate the probability of failures qualitatively.
- Step 8. Process the uncertainty in experts’ evaluations and calculate the RPN.
- Step 9. Prioritize failure modes identified with different risk levels.
- Step 10. Pay more attention to failure modes with high priorities and develop an appropriate action plan mainly from two aspects:
  - Preventive actions for avoiding a failure.
  - Compensatory actions for minimizing losses brought by an unwanted failure.
- Step 11. Make a summary of the previous analysis with tabular form.

Traditionally, during the application process of FMEA, prioritizing failure modes based on RPN is a key step. A failure mode with a higher RPN value is concerned with being more critical than that with a lower RPN.

**Definition** **6.** *The RPN consists of three factors: the probability of occurrence of a failure mode (O), the severity of a failure effect (S), and the probability of a failure being detected (D). RPN can be defined as follows:*

(9) RPN=O×S×D.

Generally, each risk factor can be measured with 10 ranking levels from 1 to 10. For instance, Table 1 shows the suggested criteria of rating for the occurrence *O* of a failure in FMEA. Similarly, the severity *S* of a failure effect and the detectability *D* of a failure can be mapped to an integer from 1 to 10. More details can be found in [46].

### 2.3. Deng Entropy

Deng entropy (DE) is proposed by Deng yong, which satisfies five axiomatic requirements: range, probabilistic consistency, set consistency, additivity, and subadditivity. Compared with Hohle’s confusion measure, Yager’s dissonance measure, and Klir and Ramer’s discord, Deng entropy can increase monotonously with the rise of the size of focal sets for mass functions in DST [41].

**Definition** **7.** *DE is defined as follows [41]:*

(10) $$DE\left(m\right)=-\sum_{A\in X}m\left(A\right)\log_{2}\frac{m\left(A\right)}{2^{\left|A\right|}-1},$$

*where m is the a mass function defined on the frame of discernment X, and A is a focal element of m. |A| stands for the cardinality of A.*

As a generalization of Shannon entropy, Deng entropy is similar with it formally. However, the belief of each focal element *A* is divided by a term, $2^{\left|A\right|}-1$, which represents the potential number of states in *A* (the empty set is not included). In addition, Deng entropy is actually a type of composite measure through a simple transformation.

**Definition** **8.** (11) $$DE\left(m\right)=\sum_{A\in X}m\left(A\right)\log_{2}\left(2^{\left|A\right|}-1\right)-m\left(A\right)\log_{2}m\left(A\right),$$

*where the term, $\sum_{A\in X}m\left(A\right)\log_{2}\left(2^{\left|A\right|}-1\right)$, can be regarded as a measure of a total nonspecificity in the mass function m, and the remainder of Equation (11), $-\sum_{A\in X}m\left(A\right)\log_{2}m\left(A\right)$, is the measure of discord of the mass function among various focal elements.*

## 3. Deng Entropy Weighted Risk Priority Number for FMEA

**Figure 2:**
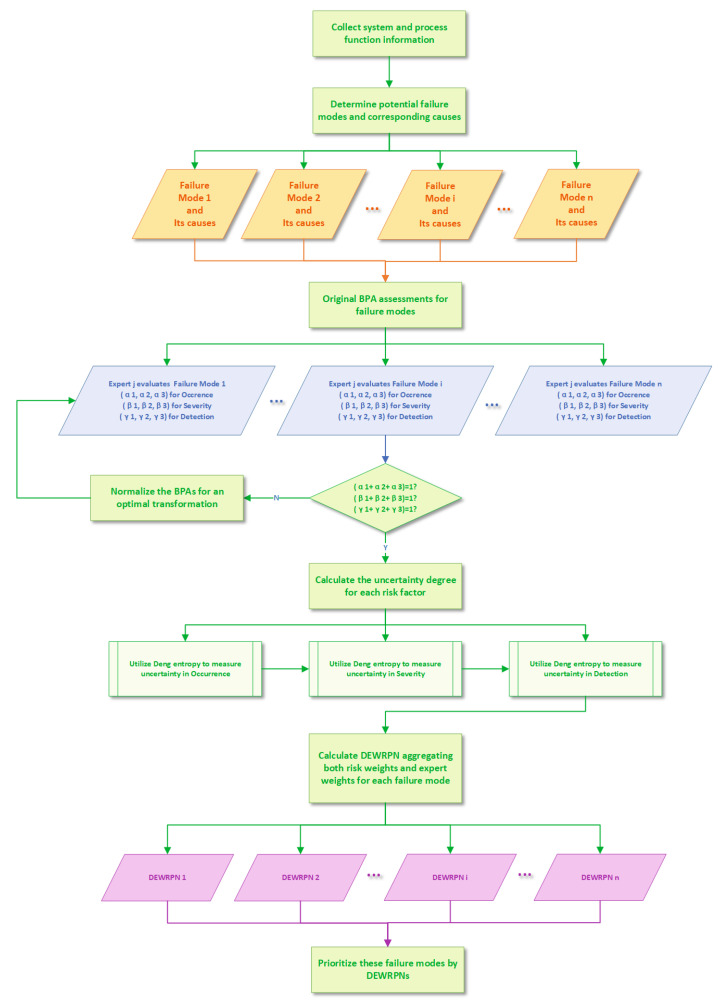
The flowchart of Deng entropy weighted risk priority number (DEWRPN) model for FMEA.

A new RPN model is proposed based on the DE in the DST framework to handle the relative weight of each risk factor and each expert in the FMEA model. The proposed method is composed of the following seven steps concluded from the new DEWRPN model for FMEA process in Figure 2:

- **Step 1.** List all failure modes (FMs) and corresponding cause of these failure modes in the system on a basis of historical data, past experiences, and expert opinions.
- **Step 2.** FMEA experts give assessments on each FMEA item for three indicators $\left(O,S,D\right)$. In DST framework, the collected subjective assessments are modeled as BPAs.
- **Step 3.** Original BPAs need to be normalized for an optimal transformation. Usually, the pignistic distribution is taken advantage of.
- **Step 4.** Measure the uncertainty degree of each risk factor by DE, which is also regarded as the risk factor weight.

**Definition** **9.** *According to the definition of Equation (10), the uncertainty degree of each risk factor for the ith expert can be calculated as follows:*

(12) $$\begin{array}{c} DE\left(O_{i}\right)=-\sum_{A\in O_{i}\subseteq X}m\left(A\right)\log_{2}\frac{m\left(A\right)}{2^{\left|A\right|}-1},\\ DE\left(S_{i}\right)=-\sum_{A\in S_{i}\subseteq X}m\left(A\right)\log_{2}\frac{m\left(A\right)}{2^{\left|A\right|}-1},\\ DE\left(D_{i}\right)=-\sum_{A\in D_{i}\subseteq X}m\left(A\right)\log_{2}\frac{m\left(A\right)}{2^{\left|A\right|}-1}, \end{array}$$

*where A is a focal element of the mass function of the mathematical expression of evaluation corresponding to the required risk factor. X is the frame of discernment of risk factors, and $X=\left\{O,S,D\right\}$. $m\left(A\right)$ is a mass function defined on the frame of discernment X. The integrated values of all the risk factors, $O_{i}$, $S_{i}$ and $D_{i}$, by the ith expert can be calculated respectively as follows:*

(13) $$\begin{array}{c} O_{i}=\sum_{j=1}^{3}R_{j}m_{j}\left(O_{i}\right),\\ S_{i}=\sum_{j=1}^{3}R_{j}m_{j}\left(S_{i}\right),\\ D_{i}=\sum_{j=1}^{3}R_{j}m_{j}\left(D_{i}\right), \end{array}$$

*where assume j=(1, 2, 3) and $R_{1}=1$, $R_{2}=2$, $R_{3}=3$. $R_{j}$ is the corresponding rating value of evaluation grades determined differently in different applications. Thus, the value range of index j and $R_{j}$ varies from case to case. $m_{j}\left(O_{i}\right)$, $m_{j}\left(S_{i}\right)$, and $m_{j}\left(D_{i}\right)$ are the mass functions of the corresponding rating values evaluated by the ith expert.*

- **Step 5.** Calculate the absolute expert weights based on corresponding uncertainty degrees of three risk factors.

**Definition** **10.** *The absolute weight for ith expert is defined as follows.*

(14) $$\omega \left(i\right)=DE\left(O_{i}\right)+DE\left(S_{i}\right)+DE\left(D_{i}\right)$$

- **Step 6.** Calculate the novel RPNs for each FM aggregating both risk factor weights and expert weights.

**Definition** **11.** *Based on the the assessments given by n (n≥1) independent experts in an FMEA team, the **Deng entropy weighted risk priority number (DEWRPN)** for each failure mode is defined as follows:*

(15) $$DEWRPN=\sum_{i=1}^{n}\frac{\omega \left(i\right)}{\sum_{i=1}^{n}\omega \left(i\right)}{O_{i}}^{e^{DE\left(O_{i}\right)}}\times {S_{i}}^{e^{DE\left(S_{i}\right)}}\times {D_{i}}^{e^{DE\left(D_{i}\right)}}$$

*where $DE\left(\cdot \right)$ measures the uncertainty degree of the assessment as BPA, which is provided by an expert to the corresponding risk factor, and $\omega \left(\cdot \right)$ weighs the absolute experts’ weights as Definition 10. Correspondingly, $O_{i}$, $S_{i}$, and $D_{i}$ respectively aggregate the ith expert’s evaluating values for each risk factor: O, S, and D. Moreover, $e^{DE\left(\cdot \right)}$ expresses the relative weight of each risk factor based on the uncertainty degree measured by Equation (12). Meanwhile, $\frac{\omega \left(i\right)}{\sum_{i=1}^{n}\omega \left(i\right)}$ is the relative weight of ith expert compared with all experts involved in FMEA.*

- **Step 7.** Prioritize these FMs by the ranking of novel RPNs.

It should be noted that, in the DEWRPN approach, the weight for each risk factor is obtained objectively and independently from relevant evaluations. It is only connected with the assessments themselves. For example, when two experts evaluate the same failure mode with different values, the weights for three factors are surely different. Besides, the DE-based expert weight is also strongly related to assessments for three risk factors, which suggests that when the same expert assesses two failure modes, if the assessments are different, the expert weights in these two failure modes are different. In other words, the assessments from different experts have no impact on other expert weights. In short, the weight, whether for three risk factors or for experts, is objective and independent. It is measured by the uncertainty degree in assessments. This is the biggest advantage of DEWRPN.

## 4. Application and Discussion

In this section, an application of DEWRPN model for FMEA is utilized to illustrate its practicability and efficiency. The result and comparisons are discussed as well.

### 4.1. Application

The practicability and effectiveness of DEWRPN model for FMEA are verified by a case study in [66]. The evaluations for ten failure modes of this case study are given by Deshpande and Modak [66]. In this section, these failure modes are processed and ranked by the proposed method. Furthermore, the priority of an FMEA item is related to their occurrence probability, severity of the related effects, and detection to each failure mode.

The aforementioned seven steps for applying DEWRPN model for FMEA are described as follows.

**Step 1.** List the FMs by the system versus three indexes as shown in Table 2.

**Step 2.** From the adopted case study, the assessments given by FMEA experts on each FMEA item for three indicators $\left(O,S,D\right)$ are presented in [67]. In the DST framework, the collected subjective assessments are modeled as BPAs. The data are shown in detail in Table 3. From the table, there are ten FMs $FM_{1}$, $FM_{1}$, …, $FM_{10}$, three criteria $\left(O,S,D\right)$, and three FMEA experts $E_{1}$, $E_{2}$, $E_{3}$. Meanwhile, each judgment is based on three evaluation grades “good”,“moderate”,“poor”. In this case study, the first expert evaluates the severity of $FM_{1}$ with a belief of 80% that $FM_{1}$ is not serious, a belief of 10% that it may take a risk of failing, and a belief of 10% that it is serious.

**Step 3.** After examination, it is worth noting that the sum of the assessed percentages for each FMEA item factor in the original data is equal to 1, which can also be described as $\alpha_{1}+\alpha_{2}+\alpha_{3}=1$. Thus, original BPAs do not need to be normalized for an optimal transformation. The data in Table 3 can be utilized directly.

**Step 4.** According to the definition of Equation (12), the uncertainty degree of each risk factor for each expert can be measured by DE. The exponential weight based on it is also regarded as the risk factor weight. In addition, when aggregating assessment rating value for each risk factor, the three evaluation grades “good”,“moderate”,“poor” are assigned with 1, 2, and 3 respectively. Thus, the advantage of Equation (13) can be adopted. The greater score of the original rating value for a factor means greater risk for this factor. To simplify the explanation of the experiment results, the *i*th FMEA item is also described as FMi. The calculation result for FMEA item FM1 is shown in Table 4.

**Step 5.** Calculate the absolute expert weights based on corresponding uncertainty degrees of three risk factors according to Equation (10).

**Step 6.** Calculate the novel RPNs for each FM aggregating both risk factor weights and expert weights utilizing Equation (15). The calculation results are presented in Table 5. The DEWRPN-based priorities for ten FMs in the sheet steel production process are sorted from high to low, shown as follows: FM4≻FM3≻FM8≻FM7≻FM1≻FM2≻FM10≻FM5≻FM6≻FM9, where ≻ means the former has a higher risk priority than the latter.

**Step 7.** Prioritize these FMs by the ranking of novel RPNs. Using the results from Table 5, analyze the results and assign finite resource to the FMEA item with high priority. In this case study, FM4 with highest RPN value is the riskiest in ten FMs. According to Table 2, more attention may be paid to the burn-out electrode and if its cooler works properly.

### 4.2. Discussion

**Figure 3:**
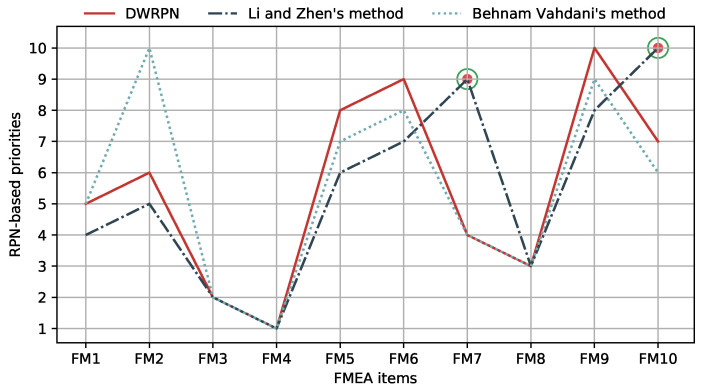
The ranking of failure modes based on the proposed method in comparison with existing methods.

In order to illustrate the validation of this novel DEWRPN model for FMEA, the results obtained for ten FMs using the proposed method are compared with the sorting results in [54,59], where the methods and experiment results are rational. The comparison result is displayed in Figure 3. Analyzing the comparison result, the ranking results yielded by DEWRPN are consistent with that obtained from Behnam Vahdani et al.’s method [54] and from Li and Chen’s method [59]. Note that, among all the 10 FMEA items, FM4 is considered to be the riskiest one, and the other failure modes have similar risk levels. Therefore, DEWRPN’s result is practical and rational.

**Figure 4:**
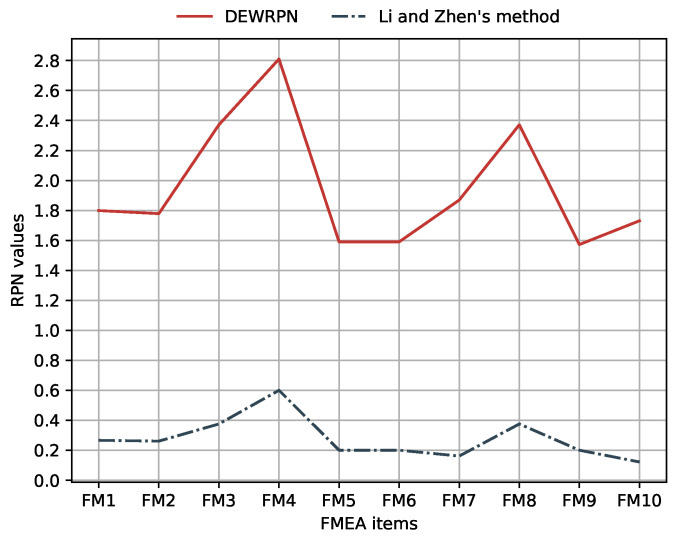
The RPN values generated by the proposed method in comparison with that of [59].

However, there exists little difference for FM7 and FM10. For FM7 and FM10, the evaluated probability distribution for the risk factors reflects the uncertainty degree, which also has an impact on risk factor weights and expert weights. Thus, FM7 and FM10 assessed by the proposed method and Beham Vahdani’s method have a higher priority than that processed by Li and Chen’s method as indicated in Figure 3. In general, compared with Behnam Vahdani et al. and Li and Chen’s method, DEWRPN model for FMEA can generate lower priorities for FM1,FM5,FM6,FM9. The reason for this can be concluded to be that the uncertainty degrees of FMEA experts’ evaluations are captured and depicted as risk factor weights and expert weights by DEWRPN. Furthermore, from the perspective of absolute RPN values, the broken line composed of RPNs obtained by Li and Chen’s method has exactly the same trend with that of the improved RPNs according to Figure 4. On the other hand, this proves the correctness of the proposed method. Moreover, by analyzing these curves, a desirable feature by which the proposed method can amplify the difference between each FM to yield a more stable and distinguishable ranking is shown.

In comparison with the aforementioned methods, DEWRPN emphasizes the corresponding weights of risk factors and absolute expert weights, which are independently obtained from the evaluations for each FMEA item. Intuitively, if the same expert evaluates two FMEA items differently, the expert weights are certainly different. This feature embodies the objectiveness of the proposed method. Considering the relative importance of both risk factors and experts, it overcomes the limitations of the traditional RPN. In addition, higher variances of RPN values of different FMs not only make RPN values more distinguishable for the prioritization process, they also make this method more applicable to some complex systems for higher accuracy in contrast to Li and Chen’s method. Last but not least, in DEWRPN, constructing RPN with risk factor weights and expert weights utilizing subjective assessments is a comprehensive way to generate RPN-based priority for each FMEA item.

In the future work, according to [3], the calculation of multiplication is meaningless on ordinal scales because the three risk factors (*O*, *S*, and *D*) are evaluated based on discrete ordinal scales of measure. So in the following work, we will consider finding an improved way to combine these three risk factors.

## 5. Conclusions

In the traditional FMEA, the shortcomings in computation of RPN have been criticized. A novel RPN model for the FMEA approach named DEWRPN is proposed in this paper. It constructs new RPN values by utilizing subjective assessments in a more comprehensive way to generate the final ranking values. Intuitively, if different experts evaluate FMEA items differently, the relative weights of experts are different. DEWRPN transforms the uncertain degree of subjective assessments, which is measured by Deng entropy, as the relative importance of risk factors and FMEA experts. A case study on the sheet steel production process verifies the practicability and efficiency of the novel method.

Future research can apply the proposed method to other industry fields. In addition, some recently proposed uncertainty measures in the evidence theory can be taken into consideration to address the different kinds of uncertainties in subjective assessments [68].

## Figures and Tables

**Table 1:** Suggested criteria of rating for occurrence of a failure in FMEA.

Rating	Probability of Occurrence	Possible Failure Rate
10	Extremely high (almost inevitable)	≥0.500
9	Very high	0.3333
8	Repeated failure	0.1250
7	High	0.0500
6	Moderately high	0.0125
5	Moderate	0.0025
4	Relatively low	0.0005
3	Low	0.0000667
2	Remote	0.0000067
1	Nearly impossible	≤0.0000066

**Table 2:** The FMEA of the sheet steel production process in Guilan steel factory.

No.	Failure Mode (FM)	Cause of Failure
$A_{1}$	Nonacceptable formation	Noncondective scrap
$A_{2}$	Nipple thread pitted	Proper coverage not obtained
$A_{3}$	Arc formation loss	Leakage of water, proper gripping loss
$A_{4}$	Burn-out electrode	Cooler not working properly
$A_{5}$	Breaking of house of pipe	Wearing of pipe due to use
$A_{6}$	Problem in movement of arm	Sever leakage
$A_{7}$	Refractory damage	Due to slag
$A_{8}$	Formation of steam	Roof leak
$A_{9}$	Refractory line damage	By hot gas
$A_{10}$	Movement of roof stop	Jam of plunger in unloader valve

**Table 3:** Group belief structure judgment of the sheet steel production process.

FMs	Experts	Severity	Occurrence	Detectability
$FM_{1}$	$E_{1}$	(0.8, 0.1, 0.1)	(0.1, 0.2, 0.7)	(0.2, 0.5, 0.3)
	$E_{2}$	(0.7, 0.0, 0.3)	(0.0, 0.4, 0.6)	(0.3, 0.4, 0.3)
	$E_{3}$	(0.8, 0.2, 0.0)	(0.1, 0.4, 0.5)	(0.2, 0.5, 0.3)
$FM_{2}$	$E_{1}$	(0.7, 0.1, 0.2)	(0.1, 0.2, 0.7)	(0.8, 0.1, 0.1)
	$E_{2}$	(0.7, 0.0, 0.3)	(0.0, 0.4, 0.6)	(0.7, 0.0, 0.3)
	$E_{3}$	(0.6, 0.4, 0.0)	(0.1, 0.4, 0.5)	(0.8, 0.2, 0.0)
$FM_{3}$	$E_{1}$	(0.8, 0.1, 0.1)	(0.0, 0.1, 0.9)	(0.2, 0.5, 0.3)
	$E_{2}$	(0.9, 0.0, 0.0)	(0.0, 0.2, 0.8)	(0.3, 0.4, 0.3)
	$E_{3}$	(0.7, 0.3, 0.0)	(0.1, 0.0, 0.9)	(0.2, 0.5, 0.3)
$FM_{4}$	$E_{1}$	(0.4, 0.4, 0.2)	(0.0, 0.1, 0.9)	(0.1, 0.2, 0.7)
	$E_{2}$	(0.3, 0.5, 0.2)	(0.0, 0.2, 0.8)	(0.0, 0.4, 0.6)
	$E_{3}$	(0.4, 0.4, 0.2)	(0.1, 0.0, 0.9)	(0.1, 0.4, 0.5)
$FM_{5}$	$E_{1}$	(0.4, 0.4, 0.2)	(0.2, 0.4, 0.4)	(0.7, 0.0, 0.3)
	$E_{2}$	(0.3, 0.5, 0.2)	(0.2, 0.4, 0.4)	(0.8, 0.2, 0.0)
	$E_{3}$	(0.4, 0.4, 0.2)	(0.1, 0.5, 0.4)	(0.6, 0.3, 0.1)
$FM_{6}$	$E_{1}$	(0.4, 0.4, 0.2)	(0.2, 0.4, 0.4)	(0.7, 0.0, 0.3)
	$E_{2}$	(0.3, 0.5, 0.2)	(0.2, 0.4, 0.4)	(0.8, 0.2, 0.0)
	$E_{3}$	(0.4, 0.4, 0.2)	(0.1, 0.5, 0.4)	(0.6, 0.3, 0.1)
$FM_{7}$	$E_{1}$	(0.4, 0.4, 0.2)	(0.2, 0.4, 0.4)	(0.1, 0.2, 0.7)
	$E_{2}$	(0.5, 0.5, 0.0)	(0.2, 0.4, 0.4)	(0.0, 0.4, 0.6)
	$E_{3}$	(0.6, 0.4, 0.0)	(0.1, 0.5, 0.4)	(0.1, 0.4, 0.5)
$FM_{8}$	$E_{1}$	(0.8, 0.1, 0.1)	(0.0, 0.1, 0.9)	(0.2, 0.5, 0.3)
	$E_{2}$	(0.9, 0.0, 0.0)	(0.0, 0.2, 0.8)	(0.3, 0.4, 0.3)
	$E_{3}$	(0.7, 0.3, 0.0)	(0.1, 0.0, 0.9)	(0.2, 0.5, 0.3)
$FM_{9}$	$E_{1}$	(0.4, 0.4, 0.2)	(0.2, 0.4, 0.4)	(0.7, 0.0, 0.3)
	$E_{2}$	(0.3, 0.5, 0.2)	(0.2, 0.4, 0.4)	(0.8, 0.2, 0.0)
	$E_{3}$	(0.4, 0.4, 0.2)	(0.1, 0.5, 0.4)	(0.6, 0.3, 0.1)
$FM_{10}$	$E_{1}$	(0.7, 0.0, 0.3)	(0.2, 0.4, 0.4)	(0.2, 0.5, 0.3)
	$E_{2}$	(0.8, 0.2, 0.0)	(0.4, 0.0, 0.6)	(0.3, 0.4, 0.3)
	$E_{3}$	(0.6, 0.3, 0.1)	(0.4, 0.0, 0.6)	(0.2, 0.5, 0.3)

**Table 4:** DE and aggregated rating values of each expert for FM1.

FM1	Expert 1	Expert 2	Expert 3
$DE\left(\cdot \right)$	$\begin{array}{c} DE\left(O_{1}\right)=1.1568\\ DE\left(S_{1}\right)=0.9219\\ DE\left(D_{1}\right)=1.4855 \end{array}$	$\begin{array}{c} DE\left(O_{2}\right)=0.9710\\ DE\left(S_{2}\right)=0.8813\\ DE\left(D_{2}\right)=1.5710 \end{array}$	$\begin{array}{c} DE\left(O_{3}\right)=1.3610\\ DE\left(S_{3}\right)=0.7219\\ DE\left(D_{3}\right)=1.4855 \end{array}$
Rating	$\begin{array}{c} O_{1}=2.6000\\ S_{1}=1.3000\\ D_{1}=2.1000 \end{array}$	$\begin{array}{c} O_{2}=2.6000\\ S_{2}=1.6000\\ D_{2}=2.0000 \end{array}$	$\begin{array}{c} O_{3}=2.4000\\ S_{3}=1.200\\ D_{3}=2.1000 \end{array}$

**Table 5:** DEWRPN values and risk priority ranking.

NO.	FM1	FM2	FM3	FM4	FM5	FM6	FM7	FM8	FM9	FM10
DEWRPN	1.7991	1.7786	2.3715	2.8101	1.5904	1.5904	1.8694	2.3715	1.5726	1.7310
Rank	5	6	2	1	8	9	4	3	10	7
